# The Efficacy of Biobrane in Managing Superficial Paediatric Burn Injuries: A Systematic Review and Meta-Analysis

**DOI:** 10.7759/cureus.78508

**Published:** 2025-02-04

**Authors:** Dost Jabarkhyl, Aude Perusseau-Lambert, Joshua Fasuyi, Mariana Kostova, Christopher Anetekhai, Dillan Mistry, Samuel Harris, Harue Yoshida, Shafiq Rahman

**Affiliations:** 1 General Internal Medicine, Luton and Dunstable University Hospital, Luton, GBR; 2 Plastic Surgery and Burns, St Andrew's Centre, Mid and South Essex National Health Service (NHS) Foundation Trust, Chelmsford, GBR; 3 Plastic and Reconstructive Surgery, Norfolk and Norwich University Hospitals National Health Service (NHS) Foundation Trust, Norwich, GBR; 4 Otolaryngology, Leeds Teaching Hospitals National Health Service (NHS) Trust, Leeds, GBR; 5 General Practice, Leeds Teaching Hospitals National Health Service (NHS) Trust, Leeds, GBR; 6 Trauma and Orthopaedics, Mid Yorkshire Hospitals National Health Service (NHS) Trust, Pinderfields Hospital, Wakefield, GBR; 7 Trauma and Orthopaedics, Royal Oldham Hospital, Greater Manchester, GBR; 8 Medicine, Doncaster and Bassetlaw Teaching Hospitals National Health Service (NHS) Foundation Trust, Doncaster, GBR; 9 Plastic and Reconstructive Surgery, Hull University Teaching Hospitals National Health Service (NHS) Trust, Hull, GBR

**Keywords:** biobrane, dressing, paediatric burn, paediatric burn injuries, superficial burn

## Abstract

Burn injuries in the paediatric population are common and account for a substantial proportion of hospital attendances, leading to a growing focus on optimising wound care to enhance healing, reduce discomfort and minimise the need for frequent dressing changes. Traditional dressings for superficial burns in children have inherent limitations that may hinder these goals. Biobrane (Dow Hickman/Bertek Pharmaceuticals, Sugar Land, TX), a semi-permeable silicone device embedded with a nylon mesh and a porcine-derived collagen matrix, offers a promising alternative with advantages such as improved wound healing, reduced pain and fewer dressing changes. This systematic review and meta-analysis, conducted in accordance with Preferred Reporting Items for Systematic Reviews and Meta-Analyses guidelines, assessed the efficacy of Biobrane by analysing data from MEDLINE, PubMed, EMCare, Cochrane Cochrane Central Register of Controlled Trials and additional clinical trial registries up to 8 June 2024. Primary outcomes included burn wound healing time, hospital length of stay and infection rate, while secondary outcomes assessed the need for split-thickness skin grafts (STSGs), pain and the number of dressing changes. Data synthesis using the OpenMetaAnalyst software (Brown University, Providence, RI) encompassed 781 burn wounds across 12 studies. The results showed that Biobrane significantly shortened wound healing time (mean difference, MD: 5.168 days, p = 0.001) and hospital length of stay (MD: 2.009 days, p < 0.001) compared to standard dressings. The infection rate was comparable (odds ratio, OR: 2.457, p = 0.132), and there was no difference in the requirement for STSGs (OR: 0.965, p = 0.956). This systematic review and meta-analysis demonstrate that Biobrane is an effective treatment for superficial paediatric burn injuries, offering faster wound healing, reduced pain and shorter hospital stays compared to traditional dressings.

## Introduction and background

Children are particularly vulnerable to burns (curiosity and exploration, lack of awareness, limited motor skills, thinner skin compared to adults, dependence on adults, and inability to escape in the event of a fire) [1]. Burns are the fifth most common cause of nonfatal paediatric injuries. While inadequate adult supervision is a major risk factor, a significant proportion of burn injuries in children are also attributed to maltreatment [1]. Children account for almost 50% of the population with severe burn injuries, and those under five years of age constitute 50%-80% of all paediatric burns [2].

Burn injuries in the paediatric population represent a significant number of urgent hospital presentations worldwide [3,4]. The majority of these injuries are scald injuries, which are superficial in nature and are usually managed conservatively [3,5]. A superficial partial thickness burn is limited to the epidermis and papillary dermis. It appears red, is painful, and generally heals within 14 days.

Optimal management of burns is crucial to prevent complications such as infection, pain, and scarring while improving patient outcomes and minimising healthcare costs associated with clinic attendance and dressing changes. Innovative wound dressings are being developed to address critical challenges in burn care, such as reducing pain, oedema, infection rates, and healing time. These advancements also aim to mitigate persistent issues, including the overuse of topical and prophylactic antibiotics, the risk of antimicrobial resistance, and optimising the frequency of dressing changes. A variety of dressings serve as valuable adjuncts in protecting superficial burn wounds and facilitating their healing process [3,4].

Silver dressings have advantages due to their antimicrobial properties. However, they may provide suboptimal epithelialisation and can be challenging in the paediatric population due to the requirement for frequent dressing changes (every three to seven days, depending on the amount of burn wound exudate), which can cause pain and distress [5]. To overcome this, various dressing materials and dermal substitutes have been developed to promote rapid healing in burn injuries and minimise patient discomfort [5-7].

Synthetic silver dressing materials are available. Acticoat (Smith & Nephew plc, London, UK), a commonly used example, has been reported to cause cytotoxic effects, which can potentially delay wound healing, requiring further skin grafting procedures [8]. Other synthetic dressing materials, for example, Suprathel (PolyMedics Innovations GmbH, Denkendorf, Germany), are utilised primarily for superficial burns, demonstrating good healing outcomes in some studies [7]. Suprathel, however, is less favourable for long-term patient comfort as it may require frequent dressing changes [7].

Since the 1980s, Porcine Xenograft (Mölnlyke, Peachtree Corners, GA) has been widely used for burn injuries with good outcomes. However, due to its biological origin, its use poses the risks of scar tissue formation and adverse immune reactions [9,10].

A synthetic dermal substitute such as Biobrane (Dow Hickman/Bertek Pharmaceuticals, Sugar Land, TX) is reported to have favourable healing outcomes, infection-resistant properties, and superior durability. It also offers long-term patient comfort as it requires fewer frequent dressing changes [11].

Biobrane is a bi-layer device composed of a silicone layer applied directly to the skin, providing a waterproof barrier, and an inner collagen matrix layer, which stimulates cell growth and helps to promote epithelisation. Biobrane was developed in the early 1970s and introduced for commercial use in 1979 [12]. Since then, it has been widely adopted in burn units and surgical centres worldwide due to its efficacy in promoting faster wound healing while maintaining patient comfort [12].

Biobrane has been studied in numerous settings and applications for the management of wounds of various aetiologies [13]. A large number of trials have studied its utilisation; however, a comprehensive systematic review and meta-analysis of randomised controlled trials and observational studies of Biobrane benefits and cost-efficiency in burns management in the paediatric population has not yet been conducted. This paper attempts to amalgamate all studies to date and enhance the current evidence base.

## Review

Methodology

A systematic review and meta-analysis were conducted according to the Preferred Reporting Items for Systematic Reviews and Meta-Analyses guidelines [14].

Eligibility Criteria

All comparative studies, including randomised and non-randomized controlled trials (RCTs) and observational studies comparing Biobrane against all other dressing types for managing superficial paediatric burns, were included. Only articles reported in English were considered to meet the eligibility criteria.

Exclusion Criteria

Studies with no comparative group, those with adult patients and where the burn depth was deep were excluded. Case reports and abstracts were also omitted from the selection process.

Literature Search Strategy

Two authors (APL and MK) independently searched the electronic databases MEDLINE, PubMed, Google Scholar, EMCare and Cochrane Central Register of Controlled Trials. The last search was run on 08 June 2024. In addition, the World Health Organization International Clinical Trials Registry (http://apps.who.int/trialsearch/), ClinicalTrials.gov (http://clinical-trials.gov/) and the International Standard Randomized Controlled Trial Number registry (http://www.isrctn.com/) were also searched to identify details of any unpublished studies. The search terms of interest included “Biobrane”, “Bio-synthetic”, “superficial burns”, “first degree burns”, “paediatric patients” and “children”. The authors also searched the reference lists of the relevant articles to optimise screening and selection.

Selection of Studies

Two authors (APL and MK) independently assessed the titles and abstracts of articles retrieved from the literature. Those meeting the eligibility criteria underwent a thorough full-text review. Studies that satisfied the criteria were subsequently selected for inclusion. Any discrepancy in study selection was resolved by a third author (SR) who acted as an adjudicator.

Data Extraction and Management

Microsoft Excel (Microsoft Corporation, Redmond, WA) was used to develop an electronic data extraction spreadsheet methodically, modified to align with Cochrane's data collection form for intervention reviews. To ensure its efficacy, the spreadsheet underwent a pilot test, during which it was used to extract data from a selection of random articles. Necessary adjustments were made to refine the tool’s functionality and accuracy. Data extraction and recording were conducted independently by two authors, APL and MK, while a third author, SR, was consulted on any uncertainties that emerged.

Data Synthesis

OpenMetaAnalyst (Brown University, Providence, RI) and Microsoft Excel were used to conduct the data synthesis. All analyses were based on a random effects model, and results were reported in forest plots with 95% confidence intervals (CIs). For continuous outcome data, the mean difference (MD) was used to assess both groups, and dichotomous outcomes were analysed with odds ratios (ORs).

Assessment of Heterogeneity

Heterogeneity among the studies was assessed using the Cochran Q test (X^2^) as well as calculating the I^2^ score, which was interpreted using the following scale: 0%-25% (low heterogeneity); 25%-75% (moderate heterogeneity); and 75%-100% (considerable heterogeneity).

Methodological Quality and Risk of Bias Assessment

The methodological quality, as well as the risk of bias for randomized control trials that met the eligibility criteria, was assessed with Cochrane’s Collaboration tool. Domains included in this evaluation were selection, performance, detection, attrition and reporting bias, as well as other sources. Studies were subsequently divided into low, unclear, or high bias. The Newcastle-Ottawa Scale assessed the methodological quality of all observational studies with domains assessing selection, comparability and exposure. The scale uses a scoring system with a maximum total score of nine stars for each study.

Outcomes

The primary outcomes were wound healing time, hospital length of stay and infection rate. The secondary outcomes included the requirement of split-thickness skin graft (STSG), pain scores and dressing changes.

Results

Literature Search Results

**Figure 1:**
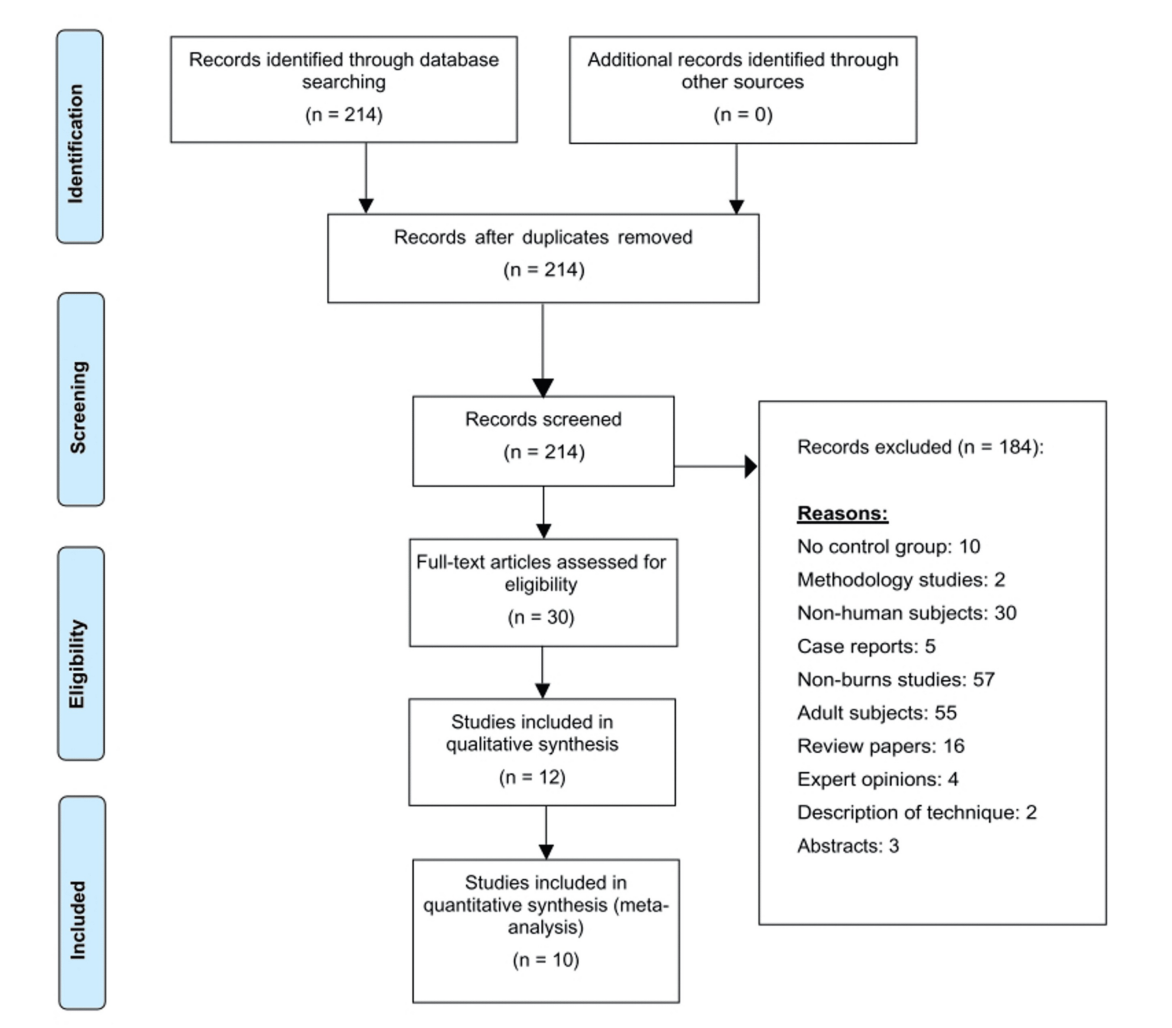
PRISMA flowchart depicting article screening and selection PRISMA: Preferred Reporting Items for Systematic Reviews and Meta-Analyses Image credit: This is an original image created by author Mariana Kostova

Two hundred and fourteen articles were retrieved, of which 12 met the eligibility criteria for systematic review and 10 for quantitative synthesis. A random effects model was used to analyse pooled data from these studies (Figure 1).

Twelve studies were selected based on eligibility criteria, and their baseline characteristics are summarised in Table 1.

**Table 1:** Amalgamation of baseline characteristics of included studies TBSA: total body surface area; N/R: not reported; BGC: beta-glucan collagen; SD: standard deviation ^*^Estimated from the graph published

Study	Study design	Mean age (years old)	Intervention dressing (n)	Control dressing (n)	Anatomical location	Mechanism of burn injury	Mean burn TBSA in % ± SD	Burn depth
Lesher et al. [11]	Retrospective cohort study	5.7 ± 5	Biobrane (n = 235)	BGC, Mölnlycke Health Care, Gothenburg, Sweden (n = 43)	Limbs, trunk (excl. face, perineum)	Scald: 194	7.6 ± 6.6	Superficial partial-thickness (n = 142) and deep partial thickness (n = 93)
						Flame: 41		
Barret et al. [15]	Randomised prospective study	3.4 ± 0.6	Biobrane (n = 10)	1% Silver sulfadiazine, Pfizer Inc., New York, NY (n = 10)	N/R	Scald: 15	8.3 ± 3	Partial thickness
						Flame: 5		
Cassidy et al. [16]	Prospective randomised controlled trial	3-18	Biobrane (n = 35)	Duoderm (n = 37)	Limbs (except hands, feet), torso, abdomen (except perineum)	Scald: 44	>10	Superficial or mid-dermal partial
						Contact: 24		
						Flame: 2		
						Others: 2		
Fan et al. [17]	Retrospective review	3.4 ± 3.6	Biobrane (n = 13)	Silver foam (Biatain Ag, Coloplast Corporation, Humlebæk, Denmark) (n = 17)	N/R	Scald: 28	10.3 ± 4.5	Superficial partial thickness burns
						Flame: 2		
Gerding et al. [18]	Randomised prospective study	20.2 ± 3.1	Biobrane (n = 26)	Silver sulfadiazine (n = 26)	N/R	Scald: 32^*^	2.2	Partial thickness
						Contact: 4^*^		
						Grease: 13^*^		
						Others: 3^*^		
Khamise et al. [19]	Retrospective comparative study	1.8 ± 7	Biobrane (n = 61)	Epiprotect (n = 38)	N/R	Scald: 98	6	Partial thickness
						Flame: 1		
Lorusso et al. [20]	Comparative study	N/R	Biobrane (n = 11)	Amniotic membrane (n = 7)	Torso, limbs	Scald: 9	6.2 ± 2.3	Superficial partial-thickness (n = 8) and deep partial thickness (n = 3)
						Flame: 2		
Hyland et al. [21]	Prospective randomised controlled pilot study	1.8	Biobrane (n = 10)	Acticoat (n = 10)	Torso, thigh, arm	Scald: 10	8	Mid-dermal
						Contact: 1		
						Flame: 1		
Kumar et al. [22]	Prospective randomised	3.6	Biobrane (n = 17)	TransCyte and Silvazine (n = 21)	N/R	Scald: 36	5	Partial thickness
Lal et al. [23]	Prospective randomised control trial	3.2 ± 0.5	Biobrane (n = 41)	Silvadene (n = 48)	Limbs, trunk	Scald: 89	11.6 ± 1	Superficial/second degree
Selvarajah et al. [24]	Retrospective	3.7	Biobrane (n = 14)	Acticoat (n = 64)	N/R	Scald: 78	Not reported	Mid-dermal
Wood et al. [25]	Randomised control trial	5	Biobrane (n = 4)	Intrasite, Acticoat, Duoderm (n = 4)	N/R	Scald: 8	6.2 ± 3	Partial thickness

Methodological Quality and Risk of Bias Assessment

The Cochrane Collaboration Tool was used to assess the risk of bias in the RCTs (Table 2), while the Newcastle-Ottawa Quality-Assessment Scale (NOS) was used to assess the risk of bias in non-randomised studies (Table 3) [26]. A NOS score of 7 is considered a good study. Overall, the quality of the non-randomised studies was good, with the exception of the study by Lorusso et al., which scored 2 stars on the NOS scale [20]. The overall risk of bias in the RCT is summarised in Table 2.

**Table 2:** Assessment of risk of bias using the modified Cochrane Collaboration tool

Study	Bias	Author’s judgment	Support for judgment
Barret et al. [15]	Random sequence generation	Low risk	Randomised but method not specified
	Allocation concealment	Unclear	Blinding method not specified
	Blinding of participants and personnel	High risk	Unable to conceal due to nature of dressing
	Blinding of outcome assessment	High risk	Unable to blind due to the nature of the study
	Incomplete outcome data	Low risk	All data included
	Selective reporting	Low risk	All outcomes reported
Cassidy et al. [16]	Random sequence generation	Low risk	Randomised but method not specified
	Allocation concealment	Unclear	Blinding method not specified
	Blinding of participants and personnel	Unclear	Blinding method not specified
	Blinding of outcome assessment	High risk	Unable to blind due to the nature of the study
	Incomplete outcome data	Low risk	All data included
	Selective reporting	Low risk	All outcomes reported
Gerding et al. [18]	Random sequence generation	Low risk	Randomization method specified
	Allocation concealment	Unclear	Blinding method not specified
	Blinding of participants and personnel	Unclear	Blinding method not specified
	Blinding of outcome assessment	High risk	Unable to blind due to the nature of the study
	Incomplete outcome data	Low risk	All data included
	Selective reporting	Low risk	All outcomes reported
Hyland et al. [21]	Random sequence generation	Low risk	Randomization method specified
	Allocation concealment	Unclear	Blinding method not specified
	Blinding of participants and personnel	Unclear	Blinding method not specified
	Blinding of outcome assessment	High risk	Unable to blind due to the nature of the study
	Incomplete outcome data	Low risk	All data included
	Selective reporting	Low risk	All outcomes reported
Kumar et al. [22]	Random sequence generation	Low risk	Randomization method specified
	Allocation concealment	Unclear	Blinding method not specified
	Blinding of participants and personnel	Unclear	Blinding method not specified
	Blinding of outcome assessment	High risk	Unable to blind due to the nature of the study
	Incomplete outcome data	Low risk	All data included
	Selective reporting	Low risk	All outcomes reported
Lal et al. [23]	Random sequence generation	Low risk	Randomization method specified
	Allocation concealment	Unclear	Blinding method not specified
	Blinding of participants and personnel	Unclear	Blinding method not specified
	Blinding of outcome assessment	High risk	Unable to blind due to the nature of the study
	Incomplete outcome data	Low risk	Adequate handling of incomplete data
	Selective reporting	Low risk	All outcomes reported
Wood et al. [25]	Random sequence generation	Low risk	Randomization method specified
	Allocation concealment	Low risk	Blinding method specified
	Blinding of participants and personnel	Low risk	Blinding method specified
	Blinding of outcome assessment	High risk	Unable to blind due to the nature of the study
	Incomplete outcome data	Low risk	All data included
	Selective reporting	Low risk	All outcomes reported

**Table 3:** Assessment of non-randomised studies using the NOS NOS: Newcastle-Ottawa Quality Assessment Scale

Study	Selection	Comparability	Outcome
Khamise et al. [19]	☆☆☆	☆☆	☆☆
Selvarajah et al. [24]	☆☆☆	☆☆	☆☆
Fan et al. [17]	☆☆☆☆	☆☆	☆☆
Lesher et al. [11]	☆☆☆	☆☆	☆☆☆
Lorusso et al. [20]	☆	☆	-

Primary Outcomes

**Figure 2:**
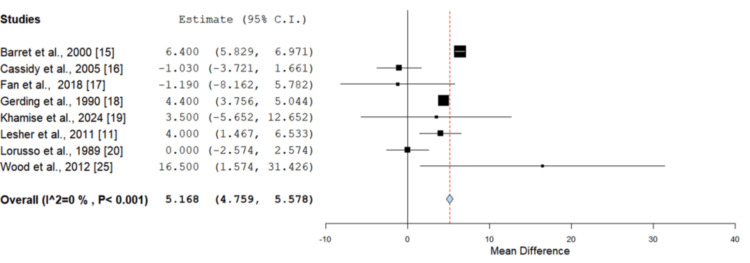
Comparison of wound healing (days) in Biobrane vs. other dressings Mean difference = 5.168 days (95% CI = 4.759-5.578), standard error = 0.209, p = 0.001. Biobrane demonstrated significantly faster wound healing compared to other dressings CI: confidence interval Image credit: This is an original image created by the author Christopher Anetekhai

Wound healing: Eight studies in total reported wound healing time in days homogeneously for Biobrane assessing it against other dressing types in superficial partial thickness paediatric burn injuries [11,12,15-20]. A significantly faster healing rate was evidenced on MD assessment in the Biobrane group (Figure 2), with a superior healing time of 5.2 days. Hyland et al. also reported a faster wound healing time in the Biobrane group (19 days) compared to Acticoat (26.5 days). However, the data did not meet the eligibility for meta-analysis, given no report of associated standard deviations (p = 0.18) [21]. This was similar to the study by Kumar et al., which identified a superior healing time for Biobrane compared to Silvazine (Smith & Nephew plc, London, UK). However, this was not amenable to a quantitative cumulative analysis [22]. TranCyte dressings, too, were assessed by Kumar et al. and identified to offer a faster re-epithelialisation rate compared to Biobrane [22]. Lal et al. reported a higher wound healing time as well per percentage burn surface area, with a significant difference seen in the Biobrane cohort compared to Silvadene (Pfizer Inc., New York, NY) [23]. Selvarajah et al. identified that the median number of days to complete healing in the Acticoat group (13 days) was faster compared to the Biobrane group (17 days). However, this difference was not statistically significant (p = 0.3) [24].

**Figure 3:**
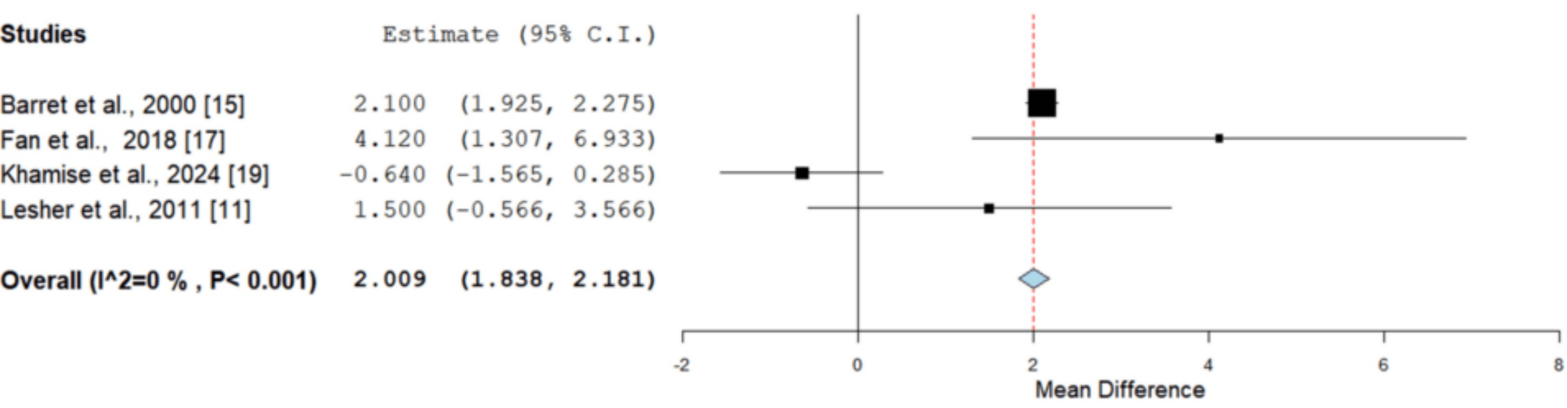
Hospital length of stay (in days) comparing Biobrane to other dressings Mean difference = 2.009 days (95% CI = 1.838-2.181), p < 0.001. The Biobrane group exhibited a significantly shorter hospital stay compared to other dressings CI: confidence interval Image credit: This is an original image created by the author Christopher Anetekhai

Hospital length of stay: Hospital length of stay was reported in four studies, which were amenable to meta-analysis, enrolling 108 patients demonstrating a significantly reduced length of stay in the Biobrane group when compared to conventional dressings (Figure 3) on MD analysis [13,15,17,19]. Lal et al. also reported similar results, with a statistically significant shorter hospital stay being observed in infants and toddlers treated with Biobrane compared to those managed with Silvadene dressing [23]. Selvarajah et al. conversely identified a prolonged hospital stay in the Biobrane group compared to Acticoat (5.5 and 2.7 days, respectively), although this was not statistically significant (p = 0.07) [24].

**Figure 4:**
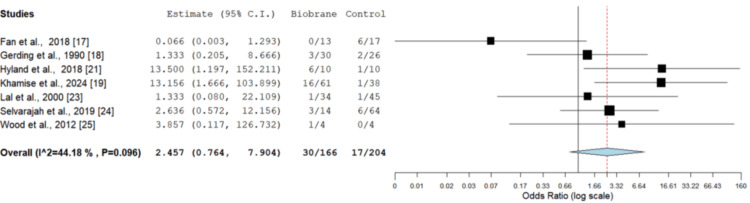
OR assessment comparing infection rate in Biobrane vs. other dressings OR = 2.457 (95% CI = 0.764-7.904), p = 0.132. No statistically significant difference in infection rates was observed between the Biobrane and other dressing groups OR: odds ratio; CI: confidence interval Image credit: This is an original image created by the author Christopher Anetekhai

Infection rate: Infection rate was reported in seven studies, including 370 burns [17-19,21,23-25]. There was no statistically significant difference between Biobrane and other dressings on OR assessment (Figure 4). The two groups were comparable (OR = 2.457, CI = 0.764-7.904, p = 0.132). A low level of heterogeneity was found among the studies (I^2^ = 0%, p = 0.77), giving further consistency to the outcome measure. No significant difference was noted regarding the infection rate between wounds dressed with Biobrane vs. other dressings (OR = 2.457, CI = 0.764-7.904, p=0.132).

Secondary Outcomes

**Figure 5:**
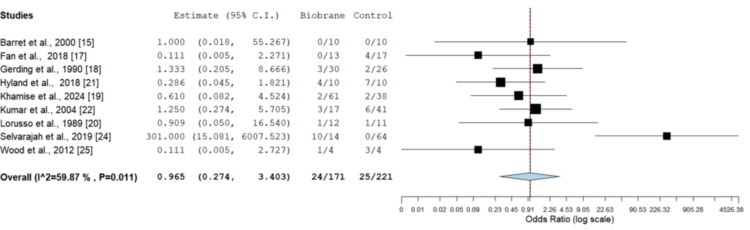
Comparison of the need for STSG in Biobrane vs. other dressings OR = 0.965 (95% CI = 0.274-3.403), p = 0.956. No significant difference was observed between the two groups STSG: split-thickness skin grafting; CI: confidence interval; OR: odds ratio Image credit: This is an original image created by the author Christopher Anetekhai

STSG requirement: The requirement of STSGs was reported in nine studies, including 392 burns (Figure 5) [15,17-22,24,25]. There was no statistically significant difference seen in the need for a skin graft to achieve healing in the Biobrane group compared with routine dressings on OR analysis (OR = 0.965, CI = 0.274-3.403, p = 0.956).

Pain scores: Pain scores were recorded in five studies. Kumar et al. showed a reduced frequency of pain medication within the Biobrane group compared to Silvazine [22]. Cassidy et al. described no significant difference in pain between Biobrane and Duoderm (ConvaTec Inc., Bridgewater, NJ) using both the Oucher and visual analogue scale pain scales, with mean aggregate scores being comparable [16]. Gerding et al. graded pain on a scale from 1 to 5 (none, minimal, mild, moderate and severe, respectively), averaging 1.6 for Biobrane and being significantly lower for silver sulfadiazine (3.6) on the first follow-up (p < 0.001) [18]. Wood et al. recorded pain on a scale of 1-10, with Biobrane showing significantly lower median pain scores than the standard treatment, consisting of Intrasite (Smith & Nephew plc, London, UK), Duoderm and Acticoat [25]. Barret et al. looked at doses of analgesic medication administered and identified a significantly reduced dependency on them in the Biobrane cohort compared to silver sulfadiazine, as well as overall pain experienced [15].

Dressing changes: Dressing changes were reported by four studies overall, with Fan et al. demonstrating a comparable frequency in the Biobrane group to silver foam [17]. This was also the case in the study by Hyland et al. when assessed against Acticoat [21]. Kumar et al. showed that Silvazine recorded significantly more dressing changes than Biobrane (9.2 vs. 2.4 p = 0.0001) [22]. Wood et al. also saw a higher number of dressing changes in their standard dressings group, consisting of Acticoat, Intrasite and Duoderm, when assessed against Biobrane with a median of 12.5 dressing changes compared to seven dressing changes in the Biobrane group [25].

Discussion

Biobrane was introduced for commercial use in 1979 [12]. It consists of a bilaminar structure with a deep layer of nylon fabric coated with Porcine-derived collagen and a superficial sealing silicon layer. The whole structure is semipermeable, and the pores allow the exchange of gases, absorption of topical antibiotics and the drainage of wound exudate. Its physical macrostructure and collagen binding attract fibrin and ingrowth of fibroblasts from the underlying wound bed, enhancing the wound healing mechanisms [14].

This combination of materials provides a temporary protective barrier that mimics the properties of natural skin. Key characteristics include protection against infection, prevention of water loss and faster healing [27]. It is easy to apply and removes itself without causing discomfort. The dressing can be trimmed away as it separates from the underlying regenerating wound bed rather than being peeled off physically from the wound during dressing changes [28].

In the present study, the authors conducted a systematic review and meta-analysis of the literature comparing Biobrane to other dressing types for superficial partial-thickness burns in children. Biobrane showed a faster healing time, shorter hospital length of stay and a comparable infection rate to other dressings used. The mean healing time and hospital stay were significantly shorter (p < 0.001) in the intervention group (Biobrane) compared to other conventional dressings (Figure 1). The infection rate was comparable between both groups (p > 0.05), as seen in Figure 3, and there was no significant difference in the requirement for split-thickness grafts (p = 0.956) either. Moderate-to-low levels of heterogeneity were seen across all quantified outcome variables, giving further consistency to the review results.

Dressing changes can be painful for people with wounds, and this triggers anxiety and stress [29]. Removing the dressing often involves peeling it off the wound, which can adhere to healing tissue and cause significant discomfort. This process can lead to distress and anxiety for young patients, potentially complicating their recovery with adverse physical and psychological sequelae associated [30]. Moreover, frequent manipulation of the wound area increases the risk of infection and disrupts the natural healing process. Therefore, the need for an ideal dressing that minimises the frequency of applications, improves healing and reduces pain for superficial partial-thickness wounds is paramount in paediatric burn care. Kumar et al. [22] showed that Silvazine required significantly more dressing changes compared to Biobrane (9.2 vs. 2.4), and this was also the case in the study by Wood et al. [25], who identified fewer dressing changes needed for Biobrane.

The use of Biobrane in paediatric patients has unique benefits in treating burn injuries. It is a biosynthetic wound dressing that effectively mimics the skin's natural barrier, reducing the risk of infection and fluid loss. This is particularly vital in children as their skin is more susceptible to dehydration and infection [31]. Biobrane also offers a less painful healing process, as it adheres to the wound, and its silicone layer peels away gently and naturally as the skin regenerates, minimising trauma and discomfort [32]. This reduces both psychological and physical distress in paediatric patients, especially the need for a potential general anaesthetic [14]. Barret et al. [15] and Gerding et al. [18] found that Biobrane had significantly lower pain scores compared to silver sulfadiazine (p < 0.001). Cassidy et al. [16] also found an improved pain experience recorded for patients with Biobrane compared to Duoderm.

Biobrane reduces healing time by preventing wound desiccation and protecting the wound surface from mechanical trauma, thereby minimising progressive dermal ischemia in the delicate zone of stasis [18]. The collagen peptides provide a scaffold for cell migration and proliferation, creating an optimal environment for wounds to heal [33]. Hyland et al. [21] found that the median time to healing was shorter in the Biobrane group compared to Acticoat; however, this was not statistically significant. Lal et al. [23] identified a significantly superior healing time compared to topical Silvadene. Cassidy et al. [16] found no difference between the healing time using Biobrane or Duoderm. Kumar et al. [22] found that TransCyte (Advanced Tissue Sciences, La Jolla, CA) enables faster healing than Biobrane; however, Biobrane was still superior to Silvazine. Epiprotect (Amnion Biosciences, Ljubljana, Slovenia) is another specialist dressing consisting of biosynthetic cellulose, which is used in the management of superficial burns, and Khamise et al. [19] conducted a direct comparison with Biobrane. Time to healing was found to be equivocal in both groups. The results of the analysis overall, however, demonstrated a significantly greater rate of re-epithelialisation when using Biobrane compared to other dressings on MD assessment by 5.2 days (Figure 2).

A reported limitation of using Biobrane is the risk of infection as it does not possess intrinsic antimicrobial properties [11]. Reported cases of Staphylococcal toxic shock syndrome after Biobrane use can cause morbidity in the paediatric population [34,35]. However, the result of the current meta-analysis demonstrates that the infection rate overall has been comparable to other dressings. Fan et al. [17] found that the Biobrane cohort had a lower infection rate compared to the silver foam cohort (p < 0.05). Lal et al. [23] found no difference in the infection rate between the Biobrane group and topical Silvadene. Compared to Epiprotect, another specialist dressing, Khamise et al. [19] identified the infection rate to be higher in the Biobrane group. The study, however, was retrospective in design, with lower numbers in the Epiprotect cohort, and the overall quantitative synthesis within this review still found the infection rate to be comparable to other dressings. This may be attributed to a number of factors, including the incorporation of Porcine collagen in Biobrane, which increases wound adherence and can therefore reduce bacterial proliferation by minimising dead space [32].

The cost of Biobrane can be a notable disadvantage, but it can prove more economical overall in terms of reducing the frequency of dressing changes and, therefore, minimising the overall financial burden [17]. This, in addition to the authors identifying a significantly lower duration of hospital stay on quantitative synthesis, can potentially mitigate the burden on hospital resources. Biobrane has high water vapour transmissibility that minimises fluid accumulations, stretchability and flexibility that facilitates application to wounds of curved or irregular surfaces, and translucency that aids in evaluating the underlying wound bed as well [36]. This overall optimises its ability to treat superficial paediatric burn injuries.

The authors conducted a systematic review and meta-analysis of 12 studies comprising 781 paediatric burn injuries managed with Biobrane dressing. Biobrane has been shown to promote a faster healing process, reducing the duration of inpatient stay, while also enhancing patient comfort through improved pain management. This quantitative review comprised RCTs and observational studies. There was low risk of bias on methodological assessment for RCTs, providing consistency in the outcome measures analysed (Table 2). Studies with observational design also scored well under the selection, comparability and exposure domains (Table 3). However, the main limitation of this review was the heterogeneity of the studies, which was mitigated using statistical methods such as the random effects model and inverse variance function. Additionally, although not all the included studies were RCTs, a significant proportion were, and the inclusion of a large total patient cohort further bolsters the reliability and validity of the findings. The authors advocate for conducting additional high-quality trials to strengthen the existing evidence base comparing Biobrane to other dressings in treating superficial paediatric burn injuries, emphasizing the need for greater consistency in the selection of comparator dressings.

## Conclusions

This study's results advocate for using Biobrane in treating superficial paediatric burn injuries. Compared to other dressings, Biobrane showed faster healing time, shorter hospital stays and a comparable infection rate. It can also reduce the frequency of dressing changes, therefore mitigating overall pain. Further high-quality trials are certainly required to enhance the current evidence base for using Biobrane for superficial paediatric burn wounds, with more consistency in comparator dressings used.
